# Balancing Fracture and Fatigue Resistance of Marshall-Designed Asphalt Mixtures with High Contents of Multi-Source Fractionated RAP

**DOI:** 10.3390/ma19153344

**Published:** 2026-08-06

**Authors:** Zeshen Jiang, Zhou Zhou, Xingyu Gu

**Affiliations:** School of Transportation, Southeast University, Nanjing 211189, China; 220243454@seu.edu.cn (Z.J.); guxingyu1976@163.com (X.G.)

**Keywords:** reclaimed asphalt pavement, balanced mix design, fracture cracking resistance, fatigue cracking resistance

## Abstract

This study examines how reclaimed asphalt pavement (RAP) dosage, virgin-binder grade, and virgin-binder content govern the cracking response of Marshall-designed mixtures. The experimental matrix comprised four RAP levels (30%, 40%, 50%, and 60%), two modified virgin binders (PG 76-22 and PG 88-34) evaluated at their respective optimum asphalt contents (OACs), and two binder-rich PG 76-22 variants (OAC + 0.3 and OAC + 0.5 percentage points); virgin mixtures served as controls. Cracking behavior was characterized by low-temperature semi-circular bending (SCB), the Illinois Flexibility Index Test (I-FIT), and direct-tension cyclic fatigue (DTCF). Fracture and fatigue indicators were subsequently integrated into cracking balance design diagrams, and k-means clustering was used to derive provisional, dataset-specific performance boundaries for long-term oven-aged mixtures. The results show that the higher-performance binder improved long-term cracking resistance at moderate RAP contents. By contrast, mixtures with 50% or 60% RAP and PG 76-22 displayed a distinctly brittle response. Increasing the PG 76-22 content above OAC produced only limited gains. These findings demonstrate that binder quality, rather than a small increase in binder dosage, is the more effective lever for balancing fracture and fatigue resistance in mixtures with substantial RAP contents.

## 1. Introduction

Reclaimed asphalt pavement (RAP) can reduce the demand for virgin materials and lower the environmental burden of asphalt production [1,2]. Its use at high dosages, however, remains constrained by specifications and by uncertainty in material consistency. This uncertainty is particularly important when reclaimed material from different projects is combined without source-based classification [3]. Many plants lack sufficient storage space to maintain numerous small, source-specific stockpiles and therefore process a heterogeneous RAP stream. Changes in source and composition can translate into fluctuations in gradation, binder content, recovered-binder properties, and ultimately, mixture performance [4,5,6,7,8]. Restrictions intended to manage this variability may leave a large proportion of the RAP accumulated at plants underused. A performance-based assessment of mixtures produced with large quantities of fractionated but otherwise unclassified RAP is therefore needed.

Size-based fractionation was therefore used as a material-management step rather than as source classification. Crushing and screening the multi-source RAP into fine (<4.75 mm) and coarse (4.75–16.0 mm) stockpiles narrow the gradation range within each feed stream, stabilize the binder content associated with each fraction, and allow the two fractions to be metered and recombined to meet the target aggregate gradation. This approach improves control of aggregate proportions and recycled-binder contribution, although it cannot eliminate source-related differences in recovered-binder properties [3,4,5,8]. The distinction is important here because this study addresses the practically relevant case of multi-source RAP that is fractionated by size but not segregated by origin. Recent comparative testing of unfractionated and progressively refined RAP streams similarly showed that fractionation reduces variability in gradation, asphalt content, and moisture, while the degree of control depends on the selected fractionation scheme [9].

A second obstacle to high RAP use is the stiff, aged binder carried by the reclaimed material [10]. Its contribution can increase mixture stiffness and reduce the ability to accommodate crack opening, and a loss of cracking resistance with RAP incorporation has been reported widely [11]. The design problem spans more than one failure mode; low-temperature fracture and cyclic fatigue at an intermediate temperature do not necessarily respond in the same way to a change in composition. A formulation that benefits one response may therefore compromise the other. Volumetric design, by itself, cannot directly resolve this trade-off or represent deterioration under prolonged traffic and environmental exposure. Balanced mix design (BMD) supplements volumetric requirements with mechanical performance criteria and offers a means of optimizing mixtures against multiple distress mechanisms [12,13,14,15,16,17]. Its conclusions nevertheless depend strongly on the selected tests and indices [14,18]. Because cracking develops through different mechanisms, no single test can be assumed to describe the full response [19]. In the present study, low-temperature SCB testing addresses fracture under quasi-static loading; I-FIT uses fracture energy together with the post-peak slope to quantify flexibility; and DTCF characterizes damage accumulation under repeated direct tension. Using these complementary methods permits the fracture-fatigue trade-off in aged, high-RAP mixtures to be examined within one BMD framework [20,21]. A remaining implementation issue is the definition of suitable acceptance limits after long-term laboratory aging [22,23]. Recent high-RAP investigations have likewise used BMD to balance cracking and rutting by adjusting recycling agents, virgin-binder grade, and binder content, and have shown that multi-level performance testing can discriminate between conventional and high-RAP mixtures [24,25,26].

Implementation studies also show that BMD criteria are test-, aging-, traffic-, and agency-specific; consequently, laboratory thresholds require local benchmarking and production validation before they are used for quality control or acceptance [12,13,14,15,16,17,18,22,23,27]. Recent work also shows that RAP thermal conditioning can affect binder interaction and the BMD-determined optimum binder content, reinforcing the need to document conditioning and performance-test selection explicitly [28].

Despite these advances, three issues remain insufficiently addressed. First, many previous studies have used RAP obtained from a single source or carefully controlled laboratory materials, whereas comparatively little is known about mixtures containing multi-source RAP that are fractionated by particle size but not classified by origin. The variability encountered in plant stockpiles may therefore not be adequately represented by the available evidence. Second, most BMD implementation studies have been developed within the Superpave design framework. In China, however, asphalt mixtures are commonly proportioned using the Marshall method [29], and the integration of complementary fracture and fatigue criteria into this design system has received limited attention. Third, existing cracking criteria are generally established for short-term-aged mixtures. The balance among low-temperature fracture, intermediate-temperature flexibility, and cyclic fatigue after long-term aging, together with suitable screening boundaries for these responses, remains unclear.

To address these gaps, this study makes three specific contributions. First, the cracking response of Marshall-designed mixtures containing 30–60% multi-source, size-fractionated RAP is evaluated while separately considering virgin-binder grade and binder dosage. Second, a cracking-oriented balance framework is constructed by combining low-temperature SCB, I-FIT, and DTCF/S-VECD results, thereby linking fracture and fatigue responses within the Marshall design procedure. Third, the relationships among the selected indices after long-term oven aging are examined, and k-means clustering is used to derive provisional, dataset-specific boundaries for classifying long-term cracking resistance. Together, these contributions provide implementation-oriented evidence for the use of high contents of multi-source RAP in surface-course mixtures.

## 2. Materials and Methods

### 2.1. Materials Evaluated in the BMD

A laboratory-produced dense-graded surface-course mixture with a nominal maximum aggregate size of 13.2 mm was selected. The RAP contents used were 0%, 30%, 40%, 50%, and 60%, with the virgin mixture used as the control. The reclaimed material came from a mixing plant in Wuxi, Jiangsu Province, where RAP from several sources was stored together. After processing, the material was divided into a fine fraction (<4.75 mm) and a coarse fraction (4.75–16.0 mm); both fractions were used in the mixture. Binder contents were 5.54% for the fine RAP and 3.26% for the coarse RAP. The binder recovered from the two fractions was classified as PG 82-10 in accordance with AASHTO M 320 [30].

Binder-grade effects were investigated with two modified virgin binders. The first was an SBS-modified PG 76-22, whereas the second, PG 88-34, contained a higher SBS dosage together with crumb rubber. Their different modification systems provided contrasting high- and low-temperature performance grades for the BMD evaluation.

All compositions were proportioned using the Marshall procedure. Laboratory Marshall specimens were prepared and compacted in accordance with ASTM D6926-20 [31] using 75 blows per face, and stability and flow were measured in accordance with ASTM D6927-22 [32]. The theoretical maximum specific gravity (Gmm) of each loose mixture and the bulk specific gravity (Gmb) of each compacted specimen were determined following ASTM D2041/D2041M-19 [33] and ASTM D2726/D2726M-21 [34], respectively. Air voids (VAs) were calculated according to ASTM D3203-22 [35]; VMA and VFA were then calculated from the measured specific gravities, aggregate specific gravity, and binder content using standard Marshall volumetric relationships. The results were checked against the Chinese highway-construction requirements, and the volumetric and Marshall results were used to establish the OAC. Table 1 summarizes the mixtures’ characteristics and combined aggregate gradations.

For the PG 76-22 series, three dosage levels were evaluated: OAC, OAC + 0.3 percentage points, and OAC + 0.5 percentage points. The latter two binder-rich variants were included to isolate the effect of an additional virgin binder; they do not represent a 0.3% or 0.5% virgin-binder replacement. PG 88-34 mixtures were evaluated at OAC only. All volumetric properties were measured using the procedures described above and are reported in Table 1.

Before performance specimens were fabricated, loose mixtures were subjected to one of two conditioning protocols. STOA used the 4 h loose-mixture procedure adopted in NCHRP Project 09-54 and earlier editions of AASHTO R 30 [36]; the material was spread approximately 38–50 mm thick and held at 135 °C for 4 h. LTOA was adapted from the 95 °C loose-mixture approach in Method C of AASHTO R 121-24 [37], which was developed from NCHRP Project 09-54; after STOA, the material remained loose in the pans at 95 °C for a fixed duration of 120 h. The pans were repositioned at evenly spaced intervals to promote uniform exposure. Unlike Method C, which selects duration from climate, pavement depth, and service period and uses a thinner loose layer, the present fixed-duration, 38–50 mm layer protocol was held constant solely for comparative laboratory conditioning. Accordingly, 120 h should not be interpreted as a universal field-aging equivalence. Aging the loose mixture before compaction provided uniformly conditioned material for the different specimen geometries used in the SCB, I-FIT, and DTCF tests [18].

### 2.2. Testing Method

#### 2.2.1. Low-Temperature SCB Test

**Figure 1:**
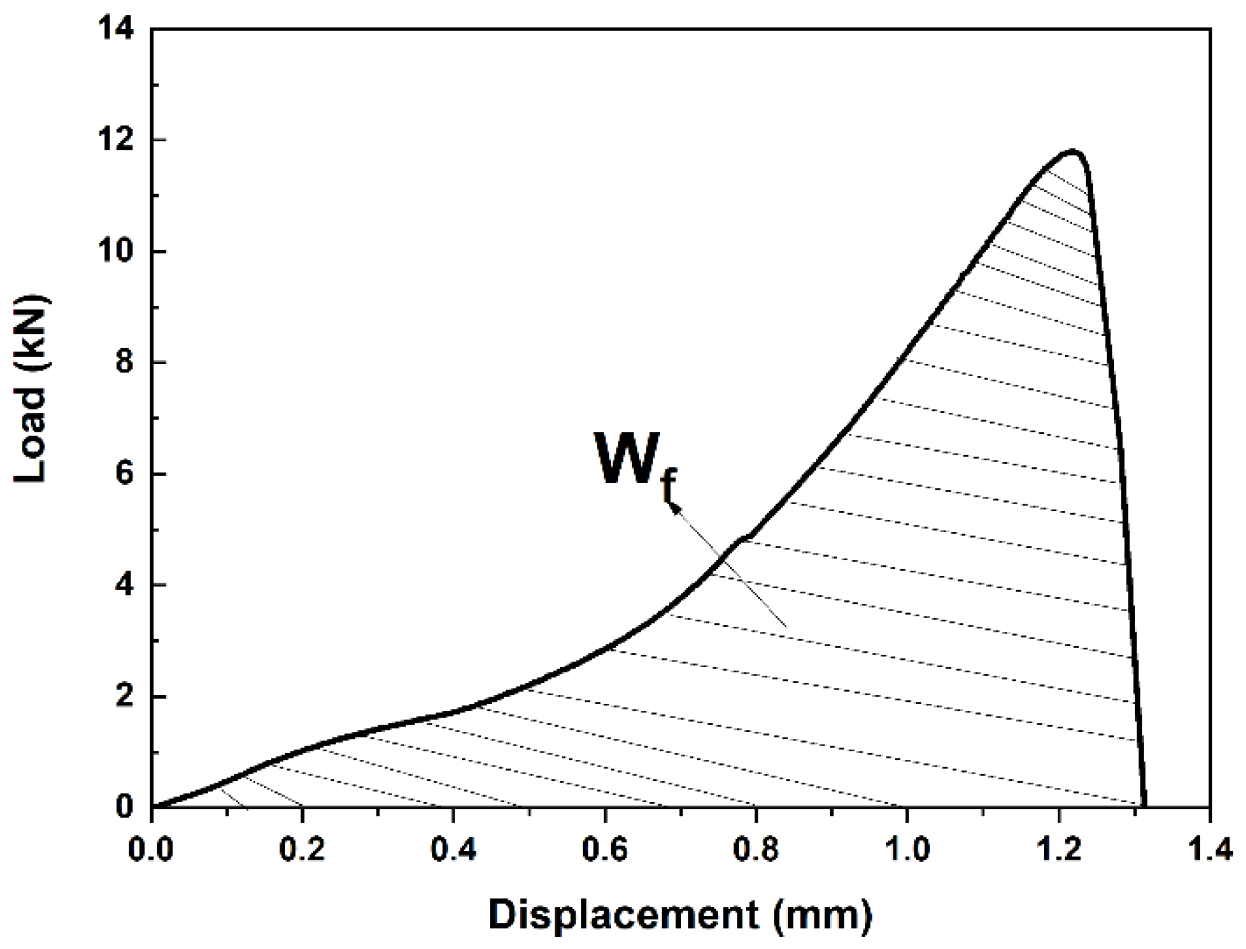
Typical curve of low-temperature SCB test.

Low-temperature SCB testing used the specimen geometry and preparation procedure in AASHTO T 394-22 [38] (formerly AASHTO TP 105-20), with a deliberate change in displacement rate from 0.03 to 50 mm/min; all specimens were conditioned and tested at −12 °C [20]. Cylindrical specimens (150 mm in diameter and 140 mm high) were compacted into 7.0% ± 0.5% air voids. Two 50 mm thick disks were cut from each cylinder and bisected along the diameter. A centrally located notch, 15 ± 1 mm deep and 2 ± 0.5 mm wide, was introduced from the flat edge of each semicircle. Specimens were then loaded vertically at constant displacement while force was recorded until complete fracture. Low-temperature resistance was expressed as fracture energy, Gf, obtained by normalizing the area beneath the load–displacement curve by the ligament area (Equation (1)); an illustrative response is provided in Figure 1.

(1) $$G_{f}=\frac{W_{f}}{A_{lig}}$$

where $G_{f}$ is the fracture energy (${J/m}^{2}$); $W_{f}$ is the work of the fracture (J); in =∫Pdu, *P* is the load and *u* is the displacement; and $A_{lig}$ is the ligament area ($m^{2}$).

#### 2.2.2. I-FIT Test

**Figure 2:**
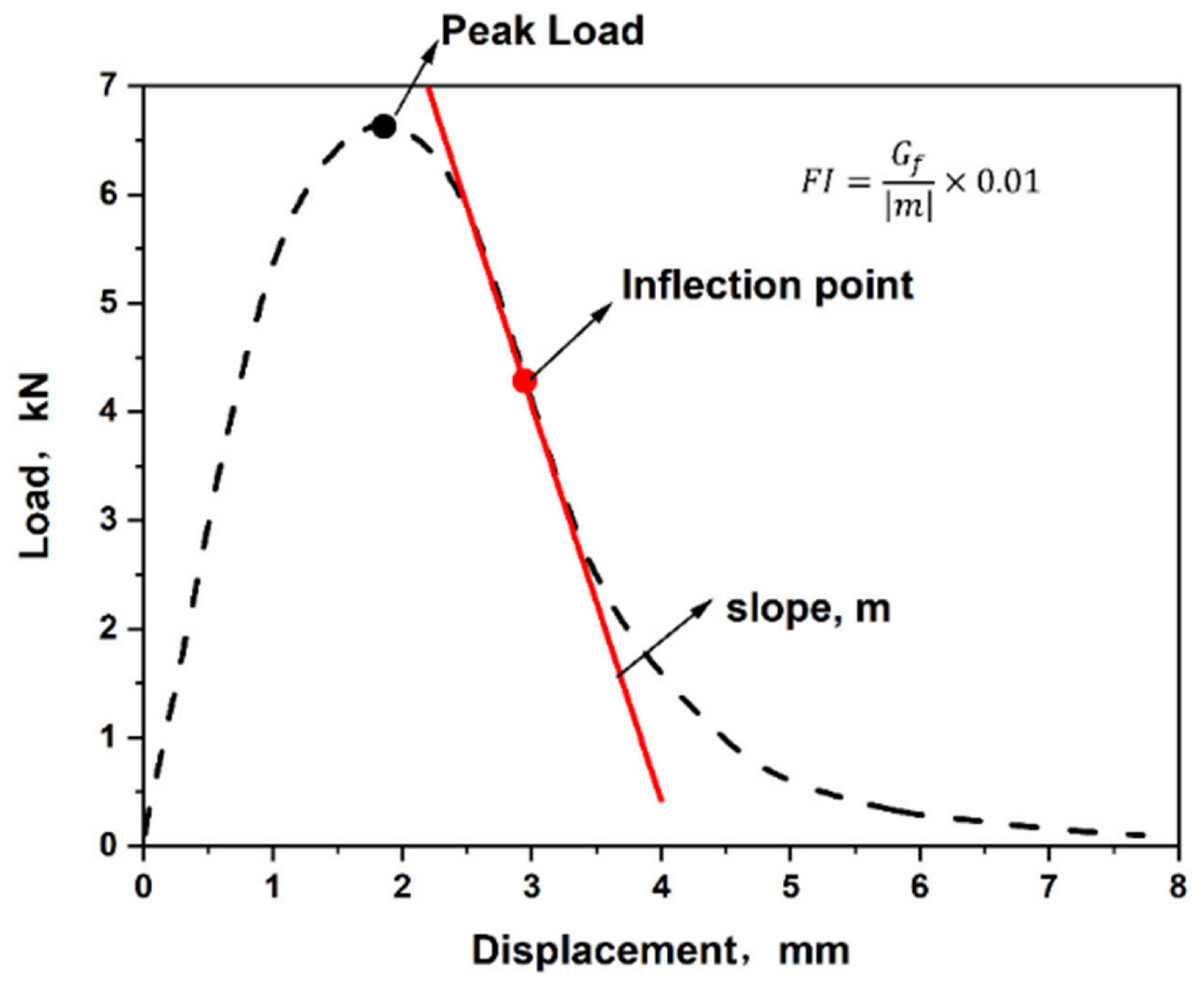
Typical curve of I-FIT test and the calculation of FI.

I-FIT was performed in the semi-circular bending configuration in accordance with AASHTO T 393-22 [39] (formerly AASHTO TP 124) [40]. Specimens were prepared using the same 150 mm diameter, 50 mm thick geometry and central-notch dimensions described for the low-temperature SCB test, and then conditioned and tested at 25 °C. A vertical displacement rate of 50 mm/min was applied, and data collection ended after the force fell below 0.5 kN. Intermediate-temperature cracking tolerance was represented by FI [41], which combines fracture energy with the absolute post-peak slope (Figure 2). A larger FI indicates that the mixture can dissipate more fracture energy while retaining a less abrupt post-peak response and is therefore more crack-tolerant; a smaller FI denotes a steeper post-peak loss of load-carrying capacity and a more brittle response.

#### 2.2.3. Direct-Tension Cyclic Fatigue Test

DTCF testing was used to quantify resistance to fatigue damage. Specimen fabrication followed AASHTO PP 99-23 [42,43]. Cylinders compacted with a Superpave gyratory compactor in 5.0% ± 0.5% air voids (150 mm diameter and 178 mm height) were cored and trimmed to the final dimensions of 38 mm by 110 mm. Viscoelastic properties were first obtained from dynamic modulus tests conducted under AASHTO T 378-22 [44] over multiple temperatures and frequencies; the measured modulus and phase angle values were used to construct master curves. Cyclic fatigue tests then followed AASHTO T 400-24 [45] (formerly AASHTO TP 133-21) [46] in an on-specimen, strain-controlled mode at 21 °C and 10 Hz. When failure was assigned to the loading cycle, the phase angle began to decrease abruptly. The DTCF records were interpreted with the simplified viscoelastic continuum damage (S-VECD) framework. In this framework, the damage characteristic curve relates pseudo-stiffness C to internal damage S and thus describes the progressive loss of integrity. Equation (2) represents this relationship with a power-law function.

(2) $$C=1-C_{11}S^{C_{12}}$$

where $C_{11}$ and $C_{12}$ are fitting coefficients for the power model.

**Figure 3:**
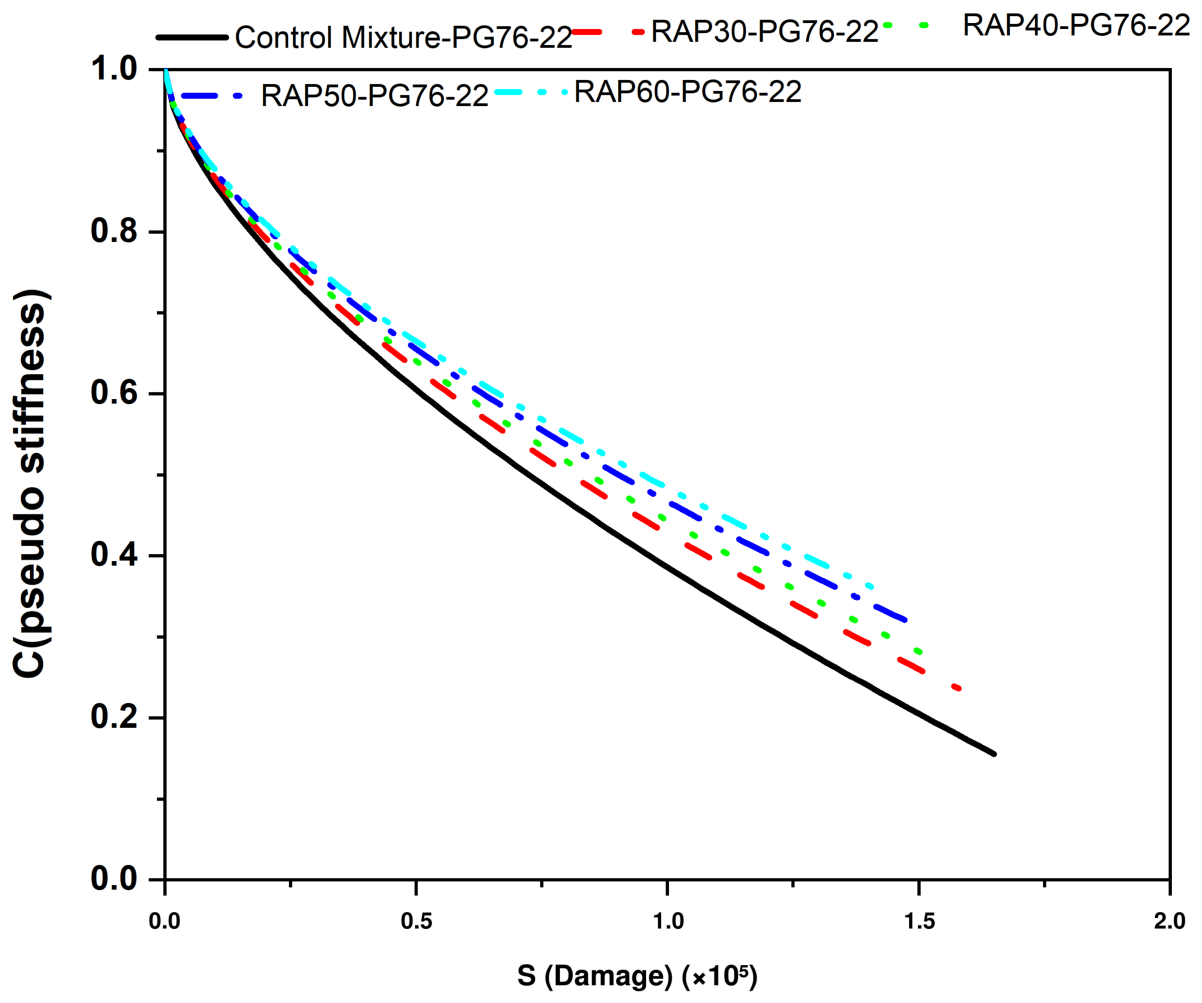
Representative pseudo-stiffness (C)–damage (S) characteristic curves for the STOA PG 76-22 series.

Figure 3 provides a representative C–S plot. The normalized pseudo-stiffness *C* is an integrity variable; *C* = 1 represents the undamaged reference state, and *C* decreases as cyclic loading accumulates the internal damage variable *S*. Thus, movement from the upper left toward the lower right of the characteristic curve represents the progressive loss of material integrity. The fitted C–S relationship, through C11 and C12, is subsequently used to calculate DR and Sapp.

Fatigue performance was additionally described by *D^R^*, the mean loss of pseudo-stiffness accumulated up to the failure cycle; its definition is given in Equation (3) [21].

(3) $$D^{R}=\frac{\int_{0}^{N_{f}}\left(1-C\right)dN}{N_{f}}$$

where $N_{f}$ is the number of cycles to failure.

The S-VECD analysis also included apparent damage capacity, *Sapp*, which combines the roles of material stiffness and toughness in fatigue response. *Sapp* was calculated with Equation (4) [47].

(4) $$S_{app}=\frac{1}{1000}\frac{{\left(\frac{{\alpha_{T}}^{\frac{C_{12}}{\alpha +1}}}{C_{11}}D^{R}\right)}^{1/C_{12}}}{{\left|E^{*}\right|}^{\alpha /4}}$$

where $\alpha_{T}$ is the time–temperature shift factor value at the target temperature, $\left|E^{*}\right|$ is the dynamic modulus (GPa) at the target temperature and 10 Hz, and $C_{11}$ and $C_{12}$ are the model coefficients.

## 3. Results and Discussion

### 3.1. Low-Temperature SCB Test Results

**Figure 4:**
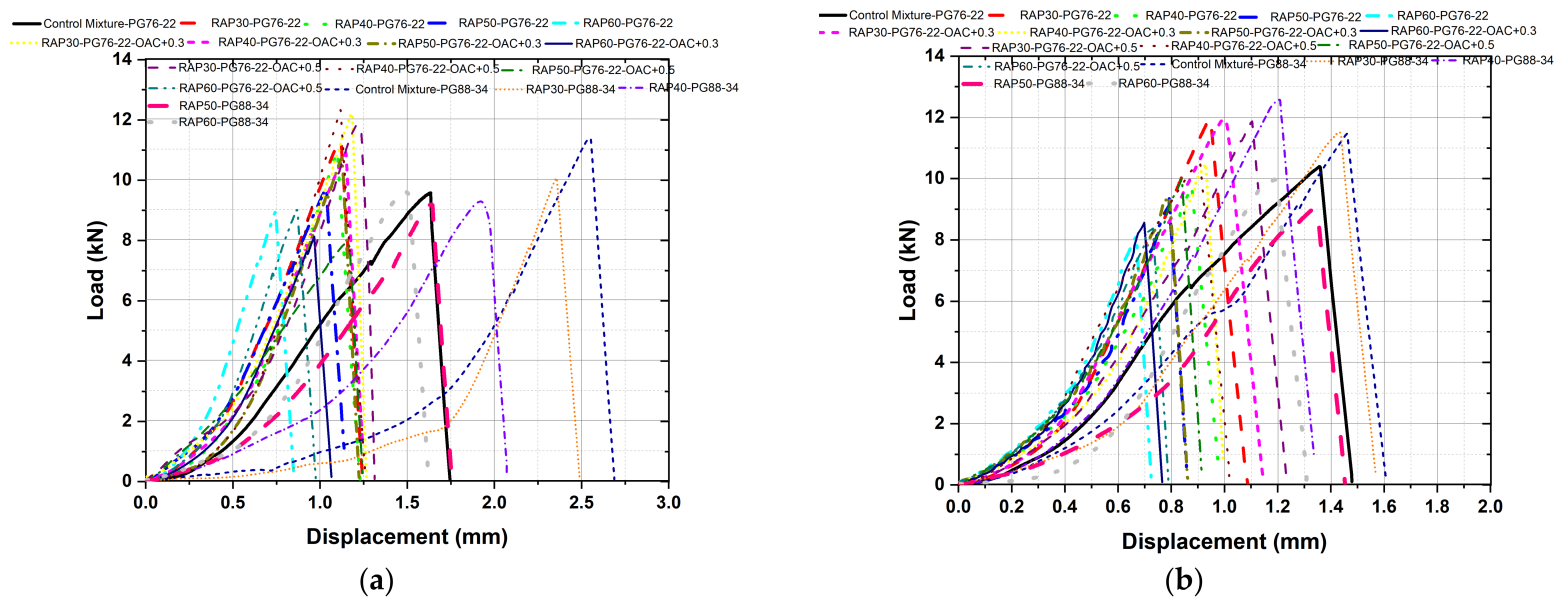
Load–displacement curves of all asphalt mixtures in low-temperature SCB test: (**a**) STOA; (**b**) LTOA.

**Figure 5:**
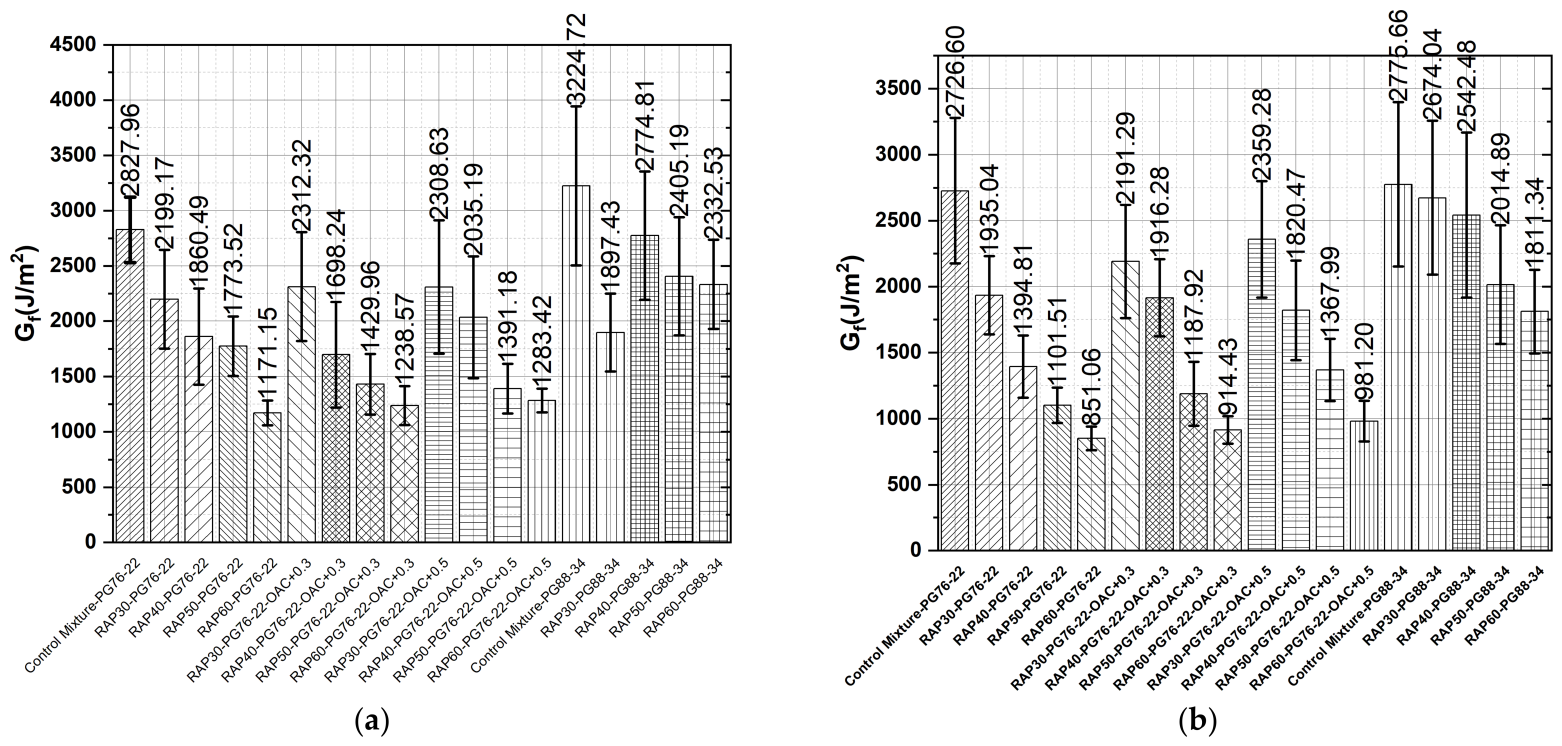
Fracture-energy results for all mixtures: (**a**) STOA; (**b**) LTOA.

The load–displacement records in Figure 4 show that all specimens were tested at −12 °C and fractured in a brittle manner; −12 °C denotes the test temperature, whereas the ordinate reports load in kN, with peak values approaching 12 kN for some mixtures. Integration of these curves yielded the fracture energies plotted in Figure 5. After STOA, fracture energy tended to decline as RAP increased, but the metric did not rise systematically with an added virgin binder. Thus, under short-term conditioning, the selected SCB response did not clearly resolve the effect of binder dosage. A more coherent composition–response relationship emerged after LTOA; higher RAP generally reduced Gf, whereas an additional virgin binder increased it in most comparisons. The two virgin control mixtures produced the strongest fracture response. Although the PG 88-34 RAP mixtures remained below the virgin controls, they generally exceeded the PG 76-22 RAP mixtures, including the variants containing extra binders. The favorable response of PG 88-34 is consistent with its lower low-temperature performance grade. Differences between the two aging conditions should nevertheless be interpreted cautiously because low-temperature SCB fracture energy can show considerable scattering; additional replicates would improve confidence in this comparison [20]. For the subsequent BMD evaluation, the LTOA fracture-energy results were therefore adopted.

### 3.2. I-FIT Test Results

**Figure 6:**
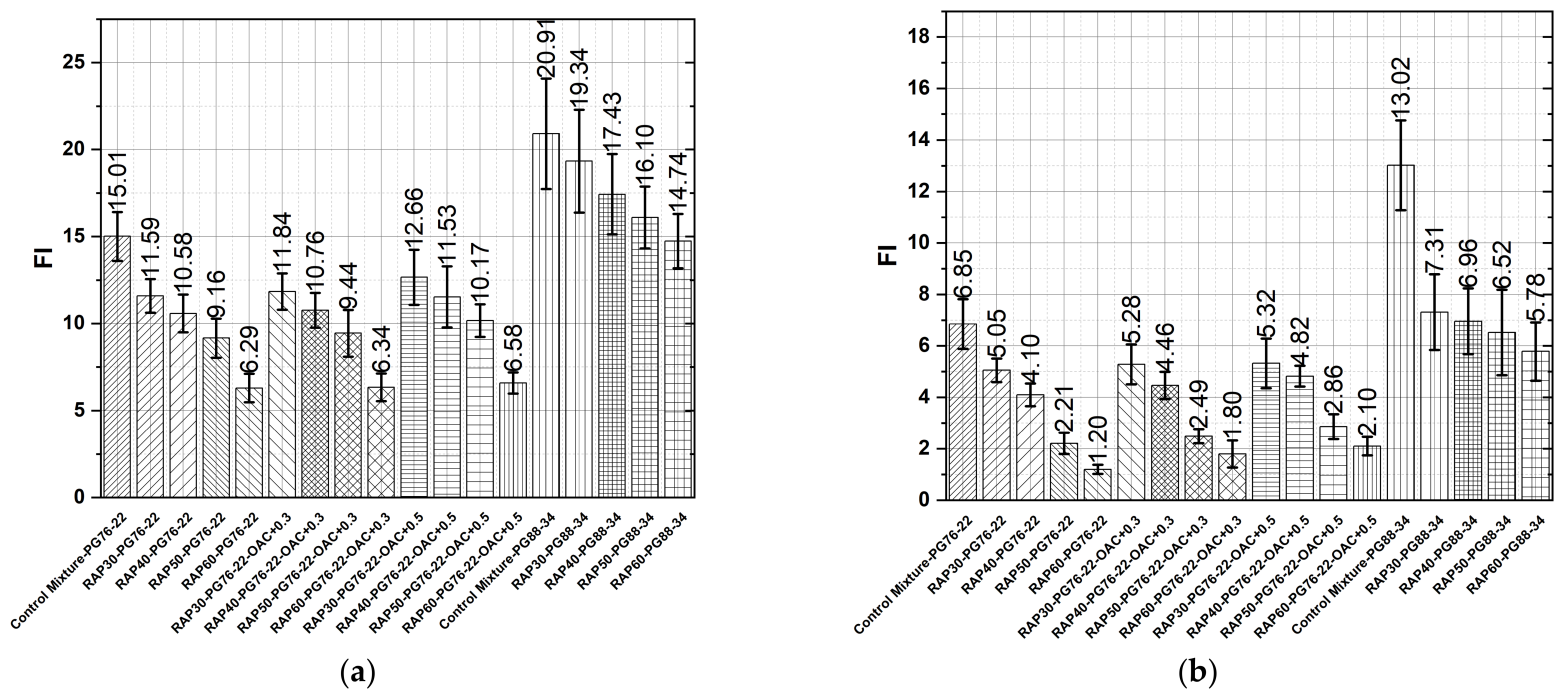
FI results of all mixtures: (**a**) STOA; (**b**) LTOA.

Figure 6 summarizes the FI measurements. FI declined monotonically with RAP dosage and was also reduced by extended aging. Adding a virgin binder produced an increase in most matched comparisons, but the magnitude was small and did not offset the effect of reclaimed material. These systematic responses confirm that I-FIT can distinguish changes in RAP content, binder dosage, and aging state. Comparing by binder grade further shows that PG 88-34 provided a substantially larger benefit than the modest increase obtained by adding more PG 76-22 binder. Binder performance grade was consequently the more influential design variable. The larger benefit from changing binder grade rather than making a small binder-content increase is consistent with recent high-RAP BMD studies that examine these design levers against both cracking and rutting performance [24,26].

For initial interpretation, FI ≥ 15 was assumed to indicate high cracking resistance and FI ≥ 8 acceptable resistance [14,27]. Under STOA, both virgin controls and the PG 88-34 mixtures containing up to 50% RAP met the high-performance level. PG 76-22 mixtures with no more than 50% RAP, together with the 60% RAP-PG 88-34 mixture, remained in the acceptable range. The PG 76-22 mixture with 60% RAP fell below FI = 8 and exhibited an overly stiff response. Aging changed this classification markedly; after LTOA, only the virgin PG 88-34 mixture approached the favorable range (FI = 13.02), while all other values were below 8. Because the published limits were not developed specifically for the adopted long-term conditioning protocol, the direct application of 15 and 8 to the LTOA dataset requires further validation.

### 3.3. DTCF Test Results

**Figure 7:**
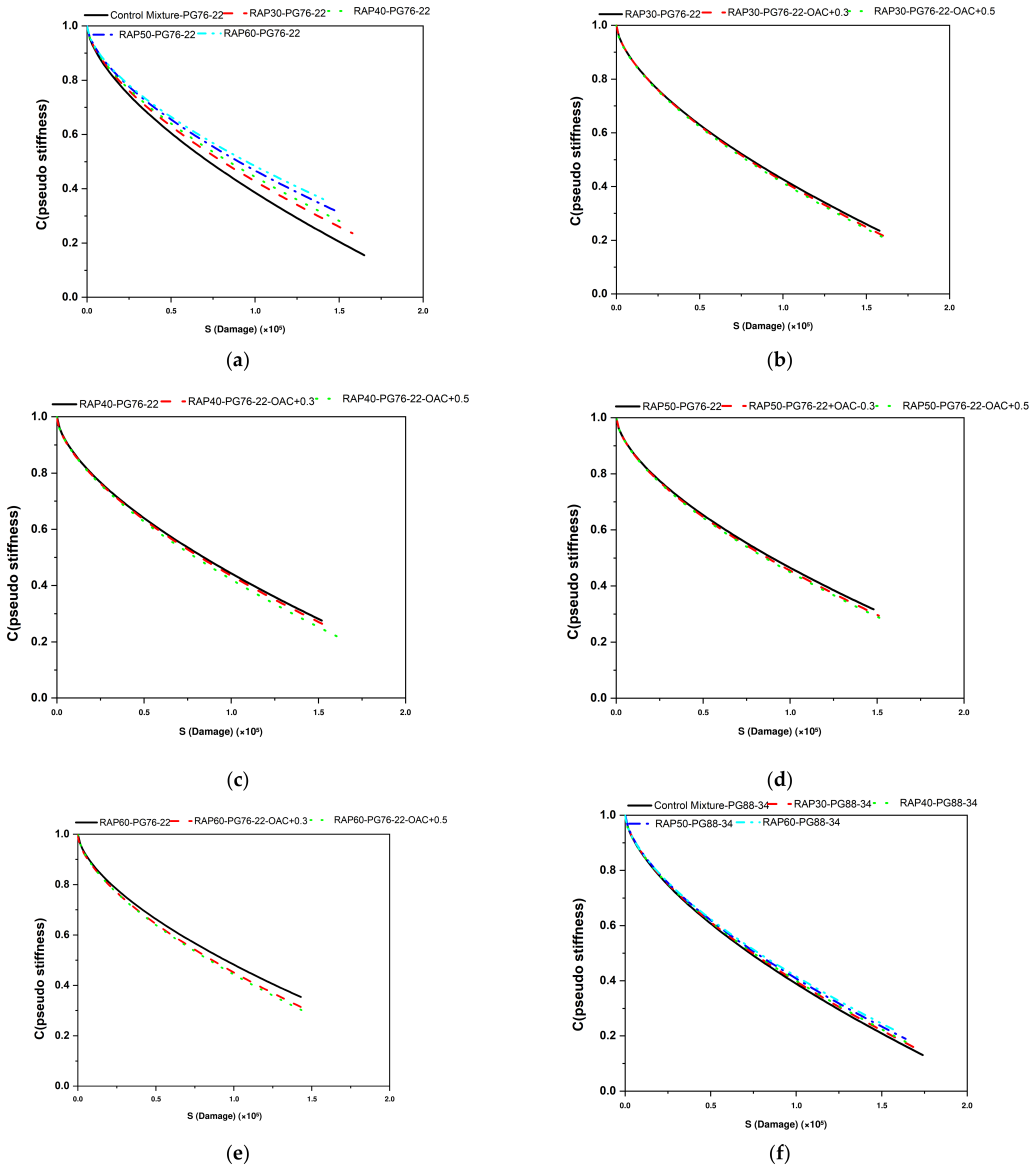
Damage characteristic curves for STOA mixtures: (**a**) RAP-content effect for PG 76-22 at OAC; (**b**–**e**) PG 76-22 binder-content effects at 30%, 40%, 50%, and 60% RAP, respectively; (**f**) RAP-content effect for PG 88-34 at OAC.

**Figure 8:**
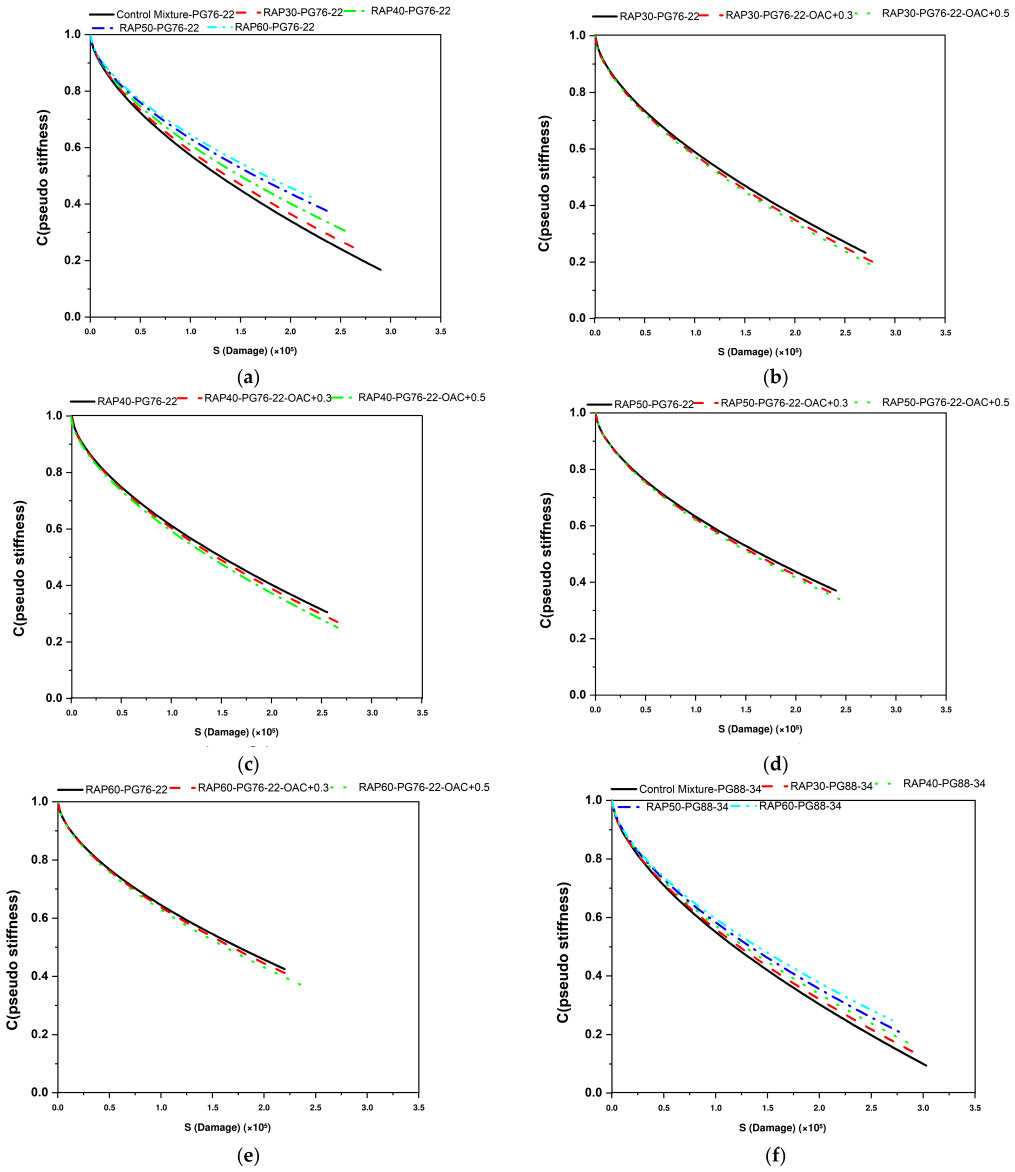
Damage characteristic curves for LTOA mixtures: (**a**) RAP-content effect for PG 76-22 at OAC; (**b**–**e**) PG 76-22 binder-content effects at 30%, 40%, 50%, and 60% RAP, respectively; (**f**) RAP-content effect for PG 88-34 at OAC.

Figure 7 and Figure 8 contain the S-VECD damage characteristic curves. Their overall shapes were comparable, but increasing RAP content or extending the aging treatment generally shifted the C–S relationship upward, a response that reflects the strong influence of modulus on curve position [21,23]. Raising the PG 76-22 virgin-binder dosage caused a smaller, but systematic, downward displacement at a given S in most matched comparisons.

At OAC, the RAP-content effect is shown for the PG 76-22 series in Figure 7a and Figure 8a and for the PG 88-34 series in Figure 7f and Figure 8f. The binder-content comparisons at 30%, 40%, 50%, and 60% RAP are shown in Figure 7b–e and Figure 8b–e, respectively. In these panels, OAC + 0.3 and OAC + 0.5 denote increases of 0.3 and 0.5 percentage points in total binder content using PG 76-22 virgin binder. At a fixed RAP level, increasing the virgin-binder content generally reduced C at a given S; the separation was modest after STOA and clearer in several LTOA comparisons, particularly at 40–60% RAP (Figure 8c–e). Because the C–S curve position is influenced by both modulus and damage evolution, this shift was interpreted together with DR and Sapp rather than as standalone evidence of longer fatigue life.

**Figure 9:**
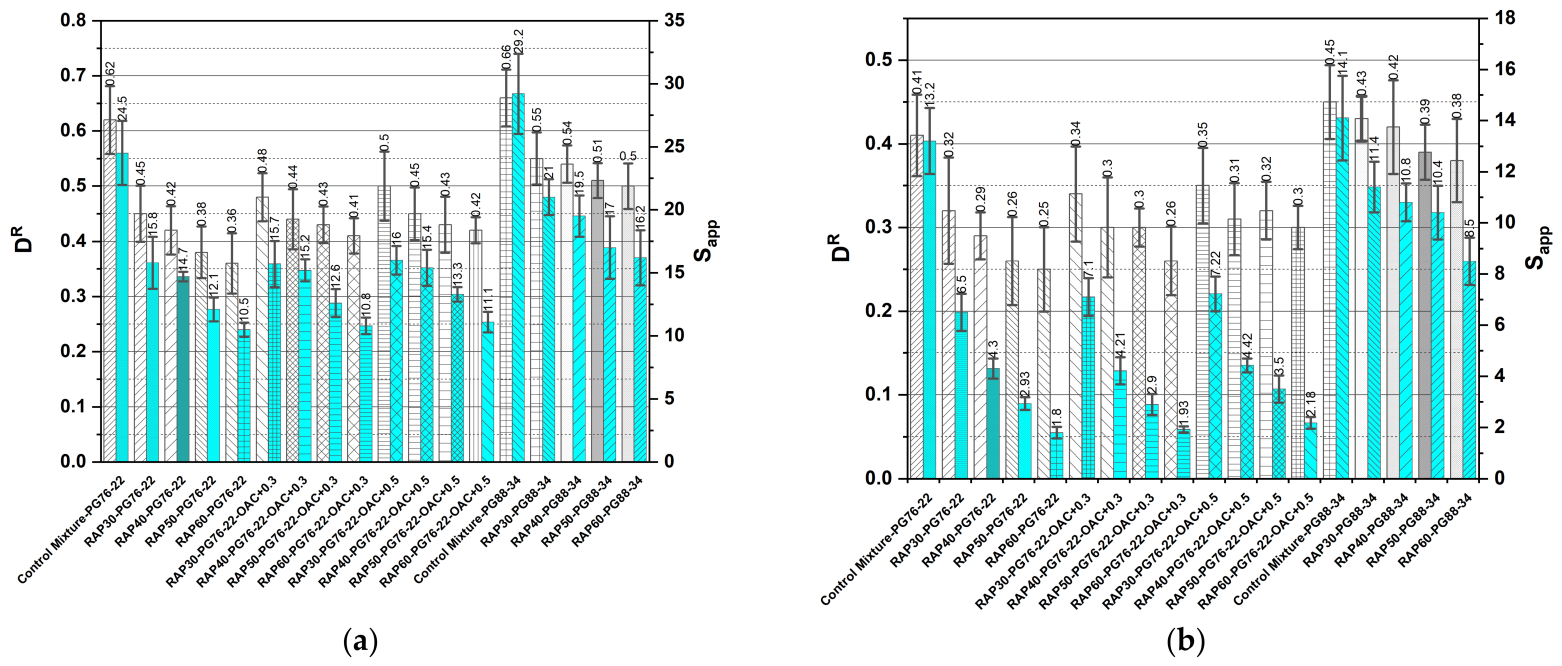
$D^{R}$ fatigue failure criterion and $S_{app}$ values for all mixtures: (**a**) STOA; (**b**) LTOA. Gray hatched bars represent $D^{R}$ (left axis), whereas cyan hatched bars represent $S_{app}$ (right axis).

The DR and Sapp results are compared in Figure 9. Both indices have been used to represent fatigue cracking resistance, with larger values associated with greater tolerance to cyclic damage [21,47,48]. In the present dataset, each index declined as RAP content or aging duration increased, demonstrating sensitivity to these two factors. Binder grade produced a clear separation; at a given RAP level, PG 88-34 mixtures generally achieved higher DR and Sapp than their PG 76-22 counterparts. The improvement is attributed to the stronger polymer-modification system of PG 88-34. Additional virgin PG 76-22 binders also increased both indices in several comparisons, but these gains were smaller than those obtained by changing to PG 88-34. The fatigue results therefore support the same design implication as I-FIT that enhancing binder quality is more effective than making a small upward adjustment to binder content.

Previous work recommends Sapp for comparative screening of mixture fatigue potential [48]. For Georgia conditions, Etheridge et al. [21] proposed minimum Sapp values of 12 for 4–10 million equivalent single-axle loads (ESALs) and 15.5 for 10–20 million ESALs. Under STOA, the two virgin controls, all PG 88-34 RAP mixtures, and the 30% RAP-PG 76-22 mixture exceeded 15.5. The remaining short-term-aged compositions fell between 8.0 and 15.5 and would therefore occupy a lower traffic category under that framework. None of the LTOA mixtures reached 15.5. These comparisons are informative, but an LTOA-specific Sapp acceptance boundary has not yet been established.

### 3.4. Correlation Between Asphalt Mixtures’ Cracking Resistance

**Figure 10:**
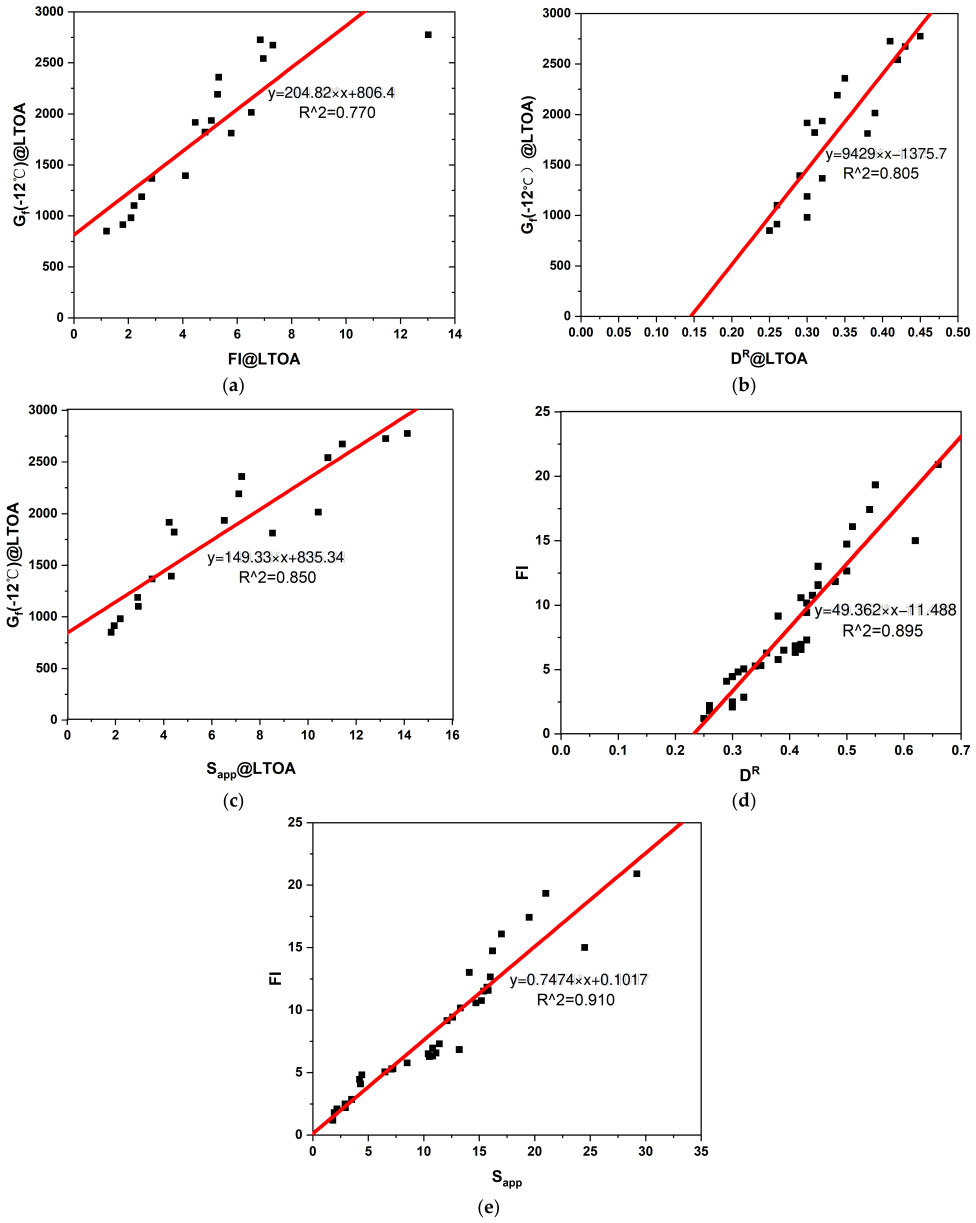
Correlation between asphalt mixture cracking resistance parameters: (**a**) $G_{f}$ vs. FI; (**b**) $G_{f}$ vs. $D^{R}$; (**c**) $G_{f}$ vs. $S_{app}$; (**d**) FI vs. $D^{R}$; (**e**) FI vs. $S_{app}$.

Pairwise regression was used to assess whether the selected indices conveyed consistent information about fracture and fatigue resistance. The analysis included low-temperature Gf, FI, DR, and Sapp. Figure 10 presents the resulting relationships, with Gf taken from the LTOA specimens. Gf and FI were well related (R^2^ = 0.770; Figure 10a). For the fatigue indices, Sapp showed stronger relationships with both Gf (R^2^ = 0.850; Figure 10c) and FI (R^2^ = 0.910; Figure 10e) than DR did (R^2^ = 0.805 and 0.895; Figure 10b and Figure 10d, respectively). This comparative correlation analysis therefore provided the explicit basis for retaining Sapp, rather than DR, in the final CBDDs. The selection does not imply that Sapp is universally superior; it reflects the stronger agreement observed within the present dataset. The correlations also suggest that a reduced set of tests may ultimately be sufficient for production acceptance, although that inference should not be extended beyond the measured range without verification. Validation with plant-produced mixtures and a broader performance range remains necessary.

### 3.5. Cracking Balance Design Diagram

**Figure 11:**
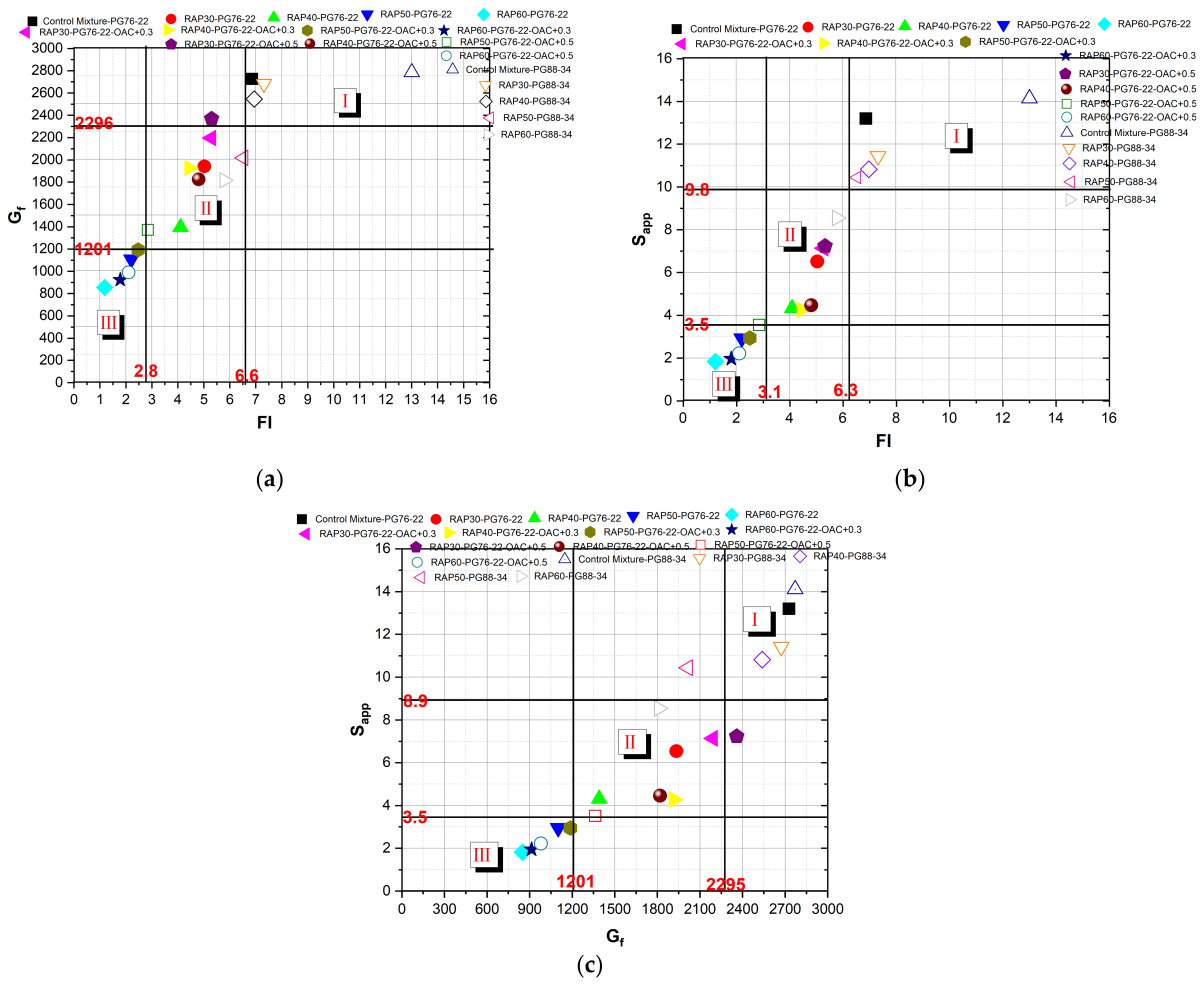
Cracking balance design diagram: (**a**) $G_{f}$ vs. FI; (**b**)FI vs. $S_{app}$; (**c**) $G_{f}$ vs. $S_{app}.$ I/II/III: high/intermediate/low resistance; color/shape: mixture composition; marker size: no data meaning.

Published short-term-aged criteria exist for FI and Sapp, but comparable limits for mixtures after long-term conditioning are not well defined. Accordingly, the LTOA mixture-level mean values of Gf, FI, and Sapp were arranged in three two-dimensional CBDDs: Gf–FI, FI–Sapp, and Gf–Sapp (Figure 11a–c). Each diagram contained one unrounded mean-data point for each of the 18 mixture compositions.

For each CBDD, the two variables were transformed to z scores before k-means clustering so that their different numerical scales did not unequally weight Euclidean distance. The number of clusters was set a priori to k = 3 to obtain three ordered screening groups. After convergence, clusters were ordered by their centroids on the two cracking indices and labeled Level I (higher resistance), Level II (intermediate resistance), and Level III (lower resistance). To express the assignments in the original engineering units, the boundary between adjacent levels on each axis was taken as the midpoint between the closest observations belonging to the neighboring clusters; the resulting values were rounded to one decimal place for FI and Sapp and to the nearest whole number for Gf. Thus, the numerical lines in Figure 11 and the ranges in Table 2 are descriptive translations of the cluster assignments, not independently calibrated specification limits.

Level I requires both paired indicators to exceed the upper diagram-specific boundaries, Level II represents the intermediate cluster between the lower and upper boundaries, and Level III represents the lowest cluster below the lower boundaries. If the two indicators placed a mixture in adjacent levels, the conservative lower level was reported; this rule assigns RAP30-PG76-22-OAC + 0.5 in the Gf–FI diagram and RAP50-PG88-34 in the Gf–Sapp diagram to Level II so that every tested composition is classified. The two virgin mixtures and the PG 88-34 mixtures with 30% or 40% RAP generally occupied Level I, whereas the PG 88-34 mixtures with 50% or 60% RAP moved toward Level II depending on the indicator pair. For PG 76-22, the 30% and 40% RAP mixtures generally occupied Level II, while the 50% and 60% RAP mixtures generally occupied Level III. Adding 0.3 or 0.5 percentage points of PG 76-22 did not materially alter the overall ranking. Because the clusters were derived from a small, correlated dataset generated within a single laboratory program, both the assignments and boundaries are sensitive to the included materials. They are therefore exploratory, dataset-specific screening categories that require independent validation with additional RAP sources, binders, mixture designs, and plant-produced materials before any use in acceptance. The need for context-specific interpretation is also consistent with recent multi-level evaluations of conventional and high-RAP BMD mixtures [25].

## 4. Conclusions

This investigation incorporated complementary fracture and fatigue tests into a BMD framework for Marshall-designed mixtures containing unclassified, fractionated RAP. Within the experimental scope, the following conclusions were drawn:

1. After LTOA, fracture energy from the −12 °C SCB test responded systematically to mixture composition. The less consistent distinction observed after STOA and the apparent influence of aging protocol on Gf warrant additional replicated testing.
2. FI from I-FIT and the DR and Sapp indices derived from DTCF/S-VECD analysis distinguished the effects of RAP dosage, virgin-binder grade, binder content, and aging. These measures are therefore suitable candidates for evaluating cracking resistance in the studied mixtures.
3. Meaningful relationships were found among low-temperature Gf, FI, and the fatigue indices, with the strongest reported association occurring between FI and Sapp. Gf, FI, and Sapp were consequently used as the complementary fracture-fatigue indicators in the balance analysis.
4. Combining the three indices in CBDDs and applying k-means clustering produced provisional cracking-resistance categories and performance boundaries for the LTOA mixtures.
5. PG 88-34 maintained high long-term cracking performance at 30% and 40% RAP. Its benefit diminished at 50% and 60% RAP, where the mixtures moved toward Level II (intermediate resistance) depending on the indicator pair. Thus, the higher-performance binder was most effective while RAP remained within a moderate range (≤40% in this experiment).
6. PG 76-22 mixtures containing 30% or 40% RAP generally fell in Level II, whereas further RAP addition promoted a stiff, brittle response and generally placed the mixtures in Level III. Raising the binder content from OAC + 0.3 to OAC + 0.5 percentage points provided little additional improvement in cracking resistance.

These conclusions are limited to the materials, conditioning procedures, and laboratory specimens evaluated here. A broader dataset covering additional RAP sources, virgin binders, mixture designs, and aging severities is needed before the observed classifications can be generalized. Plant-produced mixtures should also be examined to establish whether the laboratory trends persist during production. In particular, the proposed long-term-aging BMD thresholds require independent validation before they are used as acceptance criteria.

## Figures and Tables

**Table 1:** Summary of mixture properties and aggregate gradation.

Mixture Property	Control Mixture -PG 76-22	Control Mixture -PG 88-34	RAP30-PG 76-22	RAP40-PG 76-22	RAP50-PG 76-22	RAP60-PG 76-22	RAP30-PG 88-34	RAP40-PG 88-34	RAP50-PG 88-34	RAP60-PG 88-34	RAP30-PG 76-22-OAC + 0.3	RAP40-PG 76-22-OAC + 0.3	RAP50-PG 76-22-OAC + 0.3	RAP60-PG 76-22-OAC + 0.3	RAP30-PG 76-22-OAC + 0.5	RAP40-PG 76-22-OAC + 0.5	RAP50-PG 76-22-OAC + 0.5	RAP60-PG 76-22-OAC + 0.5
RAP content	0	0	30	40	50	60	30	40	50	60	30	40	50	60	30	40	50	60
OAC (%)	4.58	4.67	4.67	4.76	4.76	4.85	4.76	4.85	4.85	4.94	4.96	5.04	5.04	5.13	5.14	5.24	5.24	5.32
Virgin binder added content (%)	4.58	4.67	3.49	3.2	2.79	2.48	3.58	3.29	2.88	2.57	3.79	3.5	3.09	2.78	3.99	3.7	3.29	2.98
RBR (%)	0	0	25.3	32.8	41.4	48.8	24.8	32.2	40.6	47.9	23.8	30.8	39	46	22.9	29.7	37.5	44.3
Air voids (%)	4.5	4.4	4.6	4.5	4.6	4.5	4.6	4.6	4.5	4.5	4.4	4.4	4.4	4.3	4.2	4.3	4.3	4.2
VMA (%)	14.7	14.9	14.3	14	13.9	13.7	14.4	14.1	14	13.8	14.5	14.2	14.1	13.9	14.7	14.4	14.3	14.1
VFA (%)	69.4	69.8	68.5	67.9	67.6	67.2	68.8	68.1	67.9	67.4	69.0	68.3	68.1	67.6	69.4	68.8	68.5	68.1
Marshall stability (kN)	12.8	14.2	13.1	13.0	13.6	13.4	14.5	14.1	14.6	15.1	12.8	12.6	12.8	12.8	12.2	11.8	12.2	12.3
Flow value (0.1 mm)	40.4	35.1	39.5	40.3	38.2	30.8	36.2	32.5	37.5	32.0	42.2	43.0	41.5	35.2	45.1	46.8	44.6	39.1
Sieve Size, mm	Passing Percentage (%)																	
16.0	100.0	100.0	100.0	100.0	100.0	100.0	100.0	100.0	100.0	100.0	100.0	100.0	100.0	100.0	100.0	100.0	100.0	100.0
13.2	95.0	95.0	96.7	96.9	97.0	97.5	96.7	96.9	97.0	97.5	96.7	96.9	97.0	97.5	96.7	96.9	97.0	97.5
9.5	76.5	76.5	78.4	78.5	79.4	79.7	78.4	78.5	79.4	79.7	78.4	78.5	79.4	79.7	78.4	78.5	79.4	79.7
No. 4	53.0	53.0	55.0	55.5	56.0	56.4	55.0	55.5	56.0	56.4	55.0	55.5	56.0	56.4	55.0	55.5	56.0	56.4
No. 8	37.0	37.0	38.1	38.9	39.0	39.3	38.1	38.9	39.0	39.3	38.1	38.9	39.0	39.3	38.1	38.9	39.0	39.3
No. 16	26.5	26.5	27.5	27.7	27.9	28.2	27.5	27.7	27.9	28.2	27.5	27.7	27.9	28.2	27.5	27.7	27.9	28.2
No. 30	19.0	19.0	19.8	20.4	20.7	20.9	19.8	20.4	20.7	20.9	19.8	20.4	20.7	20.9	19.8	20.4	20.7	20.9
No. 50	13.5	13.5	14.3	14.6	15.0	15.4	14.3	14.6	15.0	15.4	14.3	14.6	15.0	15.4	14.3	14.6	15.0	15.4
No. 100	10.0	10.0	10.7	11.3	11.5	11.8	10.7	11.3	11.5	11.8	10.7	11.3	11.5	11.8	10.7	11.3	11.5	11.8
No. 200	6.0	6.0	6.6	6.8	7.0	7.5	6.6	6.8	7.0	7.5	6.6	6.8	7.0	7.5	6.6	6.8	7.0	7.5

OAC = optimum asphalt content; RBR = reclaimed binder ratio; VMA = voids in mineral aggregate; VFA = voids filled with asphalt.

**Table 2:** Dataset-specific LTOA cracking-resistance levels and provisional thresholds obtained from k-means clustering.

CBDD Name	Level	Mixture	Parameter 1 Threshold	Parameter 2 Threshold
$G_{f}-FI$	I	Control Mixture-PG88-34 Control Mixture-PG76-22 RAP30-PG88-34; RAP40-PG88-34	FI>6.6	$G_{f}>2296$
	II	RAP30-PG76-22; RAP40-PG76-22 RAP30-PG76-22-OAC + 0.3 RAP40-PG76-22-OAC + 0.3 RAP40-PG76-22-OAC + 0.5 RAP50-PG76-22-OAC + 0.5 RAP50-PG88-34; RAP60-PG88-34 RAP30-PG76-22-OAC + 0.5	FI>2.8<6.6	$G_{f}>1201<2296$
	III	RAP50-PG76-22; RAP60-PG76-22 RAP50-PG76-22-OAC + 0.3 RAP60-PG76-22-OAC + 0.3 RAP60-PG76-22-OAC + 0.5	FI<2.8	$G_{f}<1201$
$S_{app}-FI$	I	Control Mixture-PG76-22 Control Mixture-PG88-34 RAP30-PG88-34 RAP40-PG88-34 RAP50-PG88-34	FI>6.3	$S_{app}>9.8$
	II	RAP30-PG76-22 RAP40-PG76-22 RAP30-PG76-22-OAC + 0.3 RAP40-PG76-22-OAC + 0.3 RAP30-PG76-22-OAC + 0.5 RAP40-PG76-22-OAC + 0.5 RAP60-PG88-34	FI>3.1<6.3	$S_{app}>3.5<9.8$
	III	RAP50-PG76-22 RAP60-PG76-22 RAP50-PG76-22-OAC + 0.3 RAP60-PG76-22-OAC + 0.3 RAP50-PG76-22-OAC + 0.5 RAP60-PG76-22-OAC + 0.5	FI<3.1	$S_{app}<3.5$
$S_{app}-G_{f}$	I	Control Mixture-PG76-22 Control Mixture-PG88-34 RAP30-PG88-34 RAP40-PG88-34	$G_{f}>2295$	$S_{app}>8.9$
	II	RAP30-PG76-22 RAP40-PG76-22 RAP30-PG76-22-OAC + 0.3 RAP40-PG76-22-OAC + 0.3 RAP30-PG76-22-OAC + 0.5 RAP40-PG76-22-OAC + 0.5 RAP50-PG76-22-OAC + 0.5 RAP60-PG88-34 RAP50-PG88-34	$G_{f}>1201<2295$	$S_{app}>3.5<8.9$
	III	RAP50-PG76-22 RAP60-PG76-22 RAP50-PG76-22-OAC + 0.3 RAP60-PG76-22-OAC + 0.3 RAP60-PG76-22-OAC + 0.5	$G_{f}<1201$	$S_{app}<3.5$

## Data Availability

The original contributions presented in this study are included in the article. Further inquiries can be directed to the corresponding author.

## References

[1] Wei M., Wu S., Zhu L., Li N., Yang C. (2021). Environmental Impact on VOCs Emission of a Recycled Asphalt Mixture with a High Percentage of RAP. Materials.

[2] Tarsi G., Tataranni P., Sangiorgi C. (2020). The Challenges of Using Reclaimed Asphalt Pavement for New Asphalt Mixtures: A Review. Materials.

[3] Xiao F., Xu L., Zhao Z., Hou X. (2023). Recent Applications and Developments of Reclaimed Asphalt Pavement in China, 2010–2021. Sustain. Mater. Technol..

[4] Mocelin D.M., Isied M.M., Castorena C. (2023). Influence of Reclaimed Asphalt Pavement (RAP) and Recycled Asphalt Shingle (RAS) Binder Availability on the Composition of Asphalt Mixtures. J. Clean. Prod..

[5] Gao J., Yang J., Yu D., Jiang Y., Ruan K., Tao W., Sun C., Luo L. (2021). Reducing the Variability of Multi-Source Reclaimed Asphalt Pavement Materials: A Practice in China. Constr. Build. Mater..

[6] Jahangiri B., Majidifard H., Meister J., Buttlar W.G. (2019). Performance Evaluation of Asphalt Mixtures with Reclaimed Asphalt Pavement and Recycled Asphalt Shingles in Missouri. Transp. Res. Rec. J. Transp. Res. Board.

[7] Roja K.L., Masad E., Mogawer W. (2021). Performance and Blending Evaluation of Asphalt Mixtures Containing Reclaimed Asphalt Pavement. Road Mater. Pavement Des..

[8] Zhang K., Huchet F., Hobbs A. (2019). A Review of Thermal Processes in the Production and Their Influences on Performance of Asphalt Mixtures with Reclaimed Asphalt Pavement (RAP). Constr. Build. Mater..

[9] Zhang Y., Li J., Sun Y. (2025). Evaluation and Control of Variability in RAP Properties Through Refined Fractionation Processing Methods. Materials.

[10] Bennert T., Daniel J.S., Mogawer W. (2014). Strategies for Incorporating Higher Recycled Asphalt Pavement Percentages: Review of Implementation Trials in Northeast States. Transp. Res. Rec. J. Transp. Res. Board.

[11] Yin F., Kaseer F., Arámbula-Mercado E., Epps Martin A. (2017). Characterising the Long-Term Rejuvenating Effectiveness of Recycling Agents on Asphalt Blends and Mixtures with High RAP and RAS Contents. Road Mater. Pavement Des..

[12] Majidifard H., Rath P., Jahangiri B., Buttlar W.G. (2023). Application of Balanced Mix Design Strategies to Missouri Dense-Graded Asphalt Mixtures. Transp. Res. Rec. J. Transp. Res. Board.

[13] Diefenderfer S.D., Bowers B.F. (2019). Initial Approach to Performance (Balanced) Mix Design: The Virginia Experience. Transp. Res. Rec. J. Transp. Res. Board.

[14] Ling C., Buchanan S. (2023). Implementation Considerations of Balanced Mix Design in Practice: Recent Experience in Vermont. Road Mater. Pavement Des..

[15] Veeraragavan R.K., Nener-Plante D., Schwartz A., Tran N.H., Myers L. (2023). Balanced Mix Design Benchmarking of Asphalt Mixtures Produced in Vermont. Transp. Res. Rec. J. Transp. Res. Board.

[16] Yin F., Taylor A.J., Tran N. (2020). Performance Testing for Quality Control and Acceptance of Balanced Mix Design.

[17] Jiang L., Shen J., Wang W., Jiang L., Shen J., Wang W. (2024). Performance of High-Dose Reclaimed Asphalt Mixtures (RAPs) in Hot In-Place Recycling Based on Balanced Design. Materials.

[18] Zhou F., Steger R., Mogawer W. (2021). Development of a Coherent Framework for Balanced Mix Design and Production Quality Control and Quality Acceptance. Constr. Build. Mater..

[19] Mirzaiyanrajeh D. (2021). Balancing Fracture and Fatigue Performance in Asphalt Pavements: A Hybrid Mechanistic and Statistical Modelling Approach. Ph.D. Thesis.

[20] Jiang J., Dong Q., Ni F., Zhao Y. (2019). Effects of Loading Rate and Temperature on Cracking Resistance Characteristics of Asphalt Mixtures Using Nonnotched Semicircular Bending Tests. J. Test. Eval..

[21] Etheridge R., Wang Y., Kim S.S., Kim Y. (2019). Evaluation of Fatigue Cracking Resistance of Asphalt Mixtures Using Apparent Damage Capacity. J. Mater. Civ. Eng..

[22] Sreedhar S., Coleri E., Obaid I.A., Kumar V. (2021). Development of a Balanced Mix Design Method in Oregon to Improve Long-Term Pavement Performance. Transp. Res. Rec. J. Transp. Res. Board.

[23] Zhou F., Hu S., Newcomb D. (2020). Development of a Performance-Related Framework for Production Quality Control with Ideal Cracking and Rutting Tests. Constr. Build. Mater..

[24] Al Hatailah H., Kassem E. (2025). Balanced Mix Design for High RAP Asphalt Mixtures Prepared with Recycling Agents. Road Mater. Pavement Des..

[25] Tong B., Habbouche J., Diefenderfer S.D., Flintsch G.W. (2024). Multi-Level Performance Evaluation of BMD Surface Mixtures with Conventional and High RAP Contents: A Case Study in Virginia. Int. J. Pavement Eng..

[26] Sukhija M., Coleri E. (2025). Integrating Balanced Mix Design for High Reclaimed Asphalt Pavements in Oregon: Adjustment in Binder Content and Binder Grade. Int. J. Pavement Eng..

[27] Bowers B.F., Diefenderfer S.D., Moore N., Lynn T. (2022). Balanced Mix Design and Benchmarking: A Case Study in Establishing Performance Test Thresholds. Transp. Res. Rec. J. Transp. Res. Board.

[28] Pinheiro G., Mazzoni L.N., Lopes A.F.D., Vasconcelos K. (2025). Influence of RAP Thermal Conditioning on the Balanced Mix Design of Recycled Asphalt Mixtures. Constr. Build. Mater..

[29] Wang W., Cheng H., Sun L., Sun Y., Liu N. (2022). Multi-Performance Evaluation of Recycled Warm-Mix Asphalt Mixtures with High Reclaimed Asphalt Pavement Contents. J. Clean. Prod..

[30] (2023). Performance-Graded Asphalt Binder.

[31] (2020). Standard Practice for Preparation of Asphalt Mixture Specimens Using Marshall Apparatus.

[32] (2022). Standard Test Method for Marshall Stability and Flow of Asphalt Mixtures.

[33] (2019). Standard Test Method for Theoretical Maximum Specific Gravity and Density of Asphalt Mixtures.

[34] (2021). Standard Test Method for Bulk Specific Gravity and Density of Non-Absorptive Compacted Asphalt Mixtures.

[35] (2022). Standard Test Method for Percent Air Voids in Compacted Asphalt Mixtures.

[36] (2024). Short-Term Laboratory Conditioning of Asphalt Mixtures.

[37] (2024). Long-Term Laboratory Conditioning of Asphalt Mixtures.

[38] (2022). Determining the Fracture Energy of Asphalt Mixtures Using the Semicircular Bend Geometry (SCB).

[39] (2022). Determining the Fracture Potential of Asphalt Mixtures Using the Illinois Flexibility Index Test (I-FIT).

[40] Al-Qadi I.L., Said I.M., Ali U.M., Kaddo J.R. (2022). Cracking Prediction of Asphalt Concrete Using Fracture and Strength Tests. Int. J. Pavement Eng..

[41] Ling C., Swiertz D., Mandal T., Teymourpour P., Bahia H. (2017). Sensitivity of the Illinois Flexibility Index Test to Mixture Design Factors. Transp. Res. Rec. J. Transp. Res. Board.

[42] (2023). Preparation of Small Cylindrical Performance Test Specimens Using the Superpave Gyratory Compactor (SGC) or Field Cores.

[43] Castorena C., Underwood B.S., Kim Y.R., Lee K., Tran N.H., Taylor A. (2021). Ruggedness and Interlaboratory Studies for Asphalt Mixture Performance Tester (AMPT) Cyclic Fatigue Test: Phase I Report.

[44] (2022). Determining the Dynamic Modulus and Flow Number for Asphalt Mixtures Using the Asphalt Mixture Performance Tester (AMPT).

[45] (2024). Determining the Damage Characteristic Curve and Failure Criterion Using the Asphalt Mixture Performance Tester (AMPT) Cyclic Fatigue Test.

[46] Rahbar-Rastegar R., Huber G., Montoya M.A., Campbell C., Haddock J.E. (2022). Demonstration Project for Asphalt Performance Engineered Mixture Design Testing.

[47] Wang Y.D., Underwood B.S., Kim Y.R. (2022). Development of a Fatigue Index Parameter, Sapp, for Asphalt Mixes Using Viscoelastic Continuum Damage Theory. Int. J. Pavement Eng..

[48] Ding J., Jiang J., Ni F., Dong Q., Zhao Z. (2020). Correlation Investigation of Fatigue Indices of Fine Aggregate Matrix (FAM) and Asphalt Mixture Containing Reclaimed Asphalt Pavement Materials. Constr. Build. Mater..

